# Receptor Tyrosine Kinases Activate Canonical WNT/β-Catenin Signaling via MAP Kinase/LRP6 Pathway and Direct β-Catenin Phosphorylation

**DOI:** 10.1371/journal.pone.0035826

**Published:** 2012-04-27

**Authors:** Pavel Krejci, Anie Aklian, Marketa Kaucka, Eva Sevcikova, Jirina Prochazkova, Jan Kukla Masek, Pavol Mikolka, Tereza Pospisilova, Tereza Spoustova, MaryAnn Weis, William A. Paznekas, Joshua H. Wolf, J. Silvio Gutkind, William R. Wilcox, Alois Kozubik, Ethylin Wang Jabs, Vitezslav Bryja, Lisa Salazar, Iva Vesela, Lukas Balek

**Affiliations:** 1 Medical Genetics Institute, Cedars-Sinai Medical Center, Los Angeles, California, United States of America; 2 Department of Animal Physiology and Immunology, Institute of Experimental Biology, Masaryk University, Brno, Czech Republic; 3 Department of Cytokinetics, Institute of Biophysics AS CR, Brno, Czech Republic; 4 Department of Orthopaedics and Sports Medicine, University of Washington, Seattle, Washington, United States of America; 5 Department of Genetics and Genomic Sciences, Mount Sinai School of Medicine, New York, New York, United States of America; 6 Oral and Pharyngeal Cancer Branch, National Institutes of Health, Bethesda, Maryland, United States of America; 7 Department of Pediatrics, University of California Los Angeles School of Medicine, Los Angeles, California, United States of America; 8 Department of Psychiatry and Human Behavior, University of California Irvine, Irvine, California, United States of America; University of Washington, United States of America

## Abstract

Receptor tyrosine kinase signaling cooperates with WNT/β-catenin signaling in regulating many biological processes, but the mechanisms of their interaction remain poorly defined. We describe a potent activation of WNT/β-catenin by FGFR2, FGFR3, EGFR and TRKA kinases, which is independent of the PI3K/AKT pathway. Instead, this phenotype depends on ERK MAP kinase-mediated phosphorylation of WNT co-receptor LRP6 at Ser1490 and Thr1572 during its Golgi network-based maturation process. This phosphorylation dramatically increases the cellular response to WNT. Moreover, FGFR2, FGFR3, EGFR and TRKA directly phosphorylate β-catenin at Tyr142, which is known to increase cytoplasmic β-catenin concentration via release of β-catenin from membranous cadherin complexes. We conclude that signaling via ERK/LRP6 pathway and direct β-catenin phosphorylation at Tyr142 represent two mechanisms used by various receptor tyrosine kinase systems to activate canonical WNT signaling.

## Introduction

The receptor tyrosine kinase (RTK) and WNT/β-catenin signaling systems represent two major routes for cellular communication that synergistically regulate many essential developmental and regenerative processes, but the mechanisms of their cross-talk remain poorly defined. Inactivation of glycogen synthase kinase 3 (GSK3) is a critical event in WNT/β-catenin signal transduction as GSK3-mediated phosphorylation of β-catenin targets it for degradation [1]. Independent of the WNT-mediated GSK3 inactivation, the phosphatidylinositol 3-kinase (PI3K)/AKT pathway also inactivates GSK3, via direct AKT-mediated phosphorylation of Ser21/9 (for GSK3α/β) [2]. As many RTK systems signal via the PI3K/AKT pathway it is believed that RTKs facilitate WNT/β-catenin signaling by PI3K/AKT-mediated GSK3 inhibition [3].

In contrast to this simple hypothesis, experimental evidence argues that the PI3K/AKT pathway does not activate WNT/β-catenin. Ectopic AKT activation or insulin treatment (which signals via AKT-mediated GSK3 inhibition), both fail to activate WNT/β-catenin signaling [4], [5]. Mice carrying alanine substitution in Ser21/9 of GSK3α/β develop without any WNT-related abnormalities [6]. Finally, the AXIN-associated GSK3 was recently shown not accessible to AKT, thus preventing cross-talk between the PI3K/AKT and WNT/β-catenin pathways via AKT-mediated GSK3 phosphorylation [7]. According to the present stage of knowledge, two pools of GSK3 exist in cells, one associated with AXIN and refractory to AKT-mediated Ser21/9 phosphorylation, and another that is inhibited by AKT [1].

In light of these data, the exact mechanism of RTK and WNT/β-catenin signaling cross-talk remains an open question. We recently demonstrated that ERK MAP kinase activates WNT/β-catenin signaling via phosphorylation of WNT co-receptor low density lipoprotein receptor-related protein 6 (LRP6) [8]. Here, we show that various RTK signaling systems activate WNT/β-catenin signaling in cells, and that this cross-talk is not mediated by PI3K/AKT. Instead, RTKs utilize ERK/LRP6 pathway and a direct phosphorylation of β-catenin to activate WNT/β-catenin signaling.

**Figure 1:**
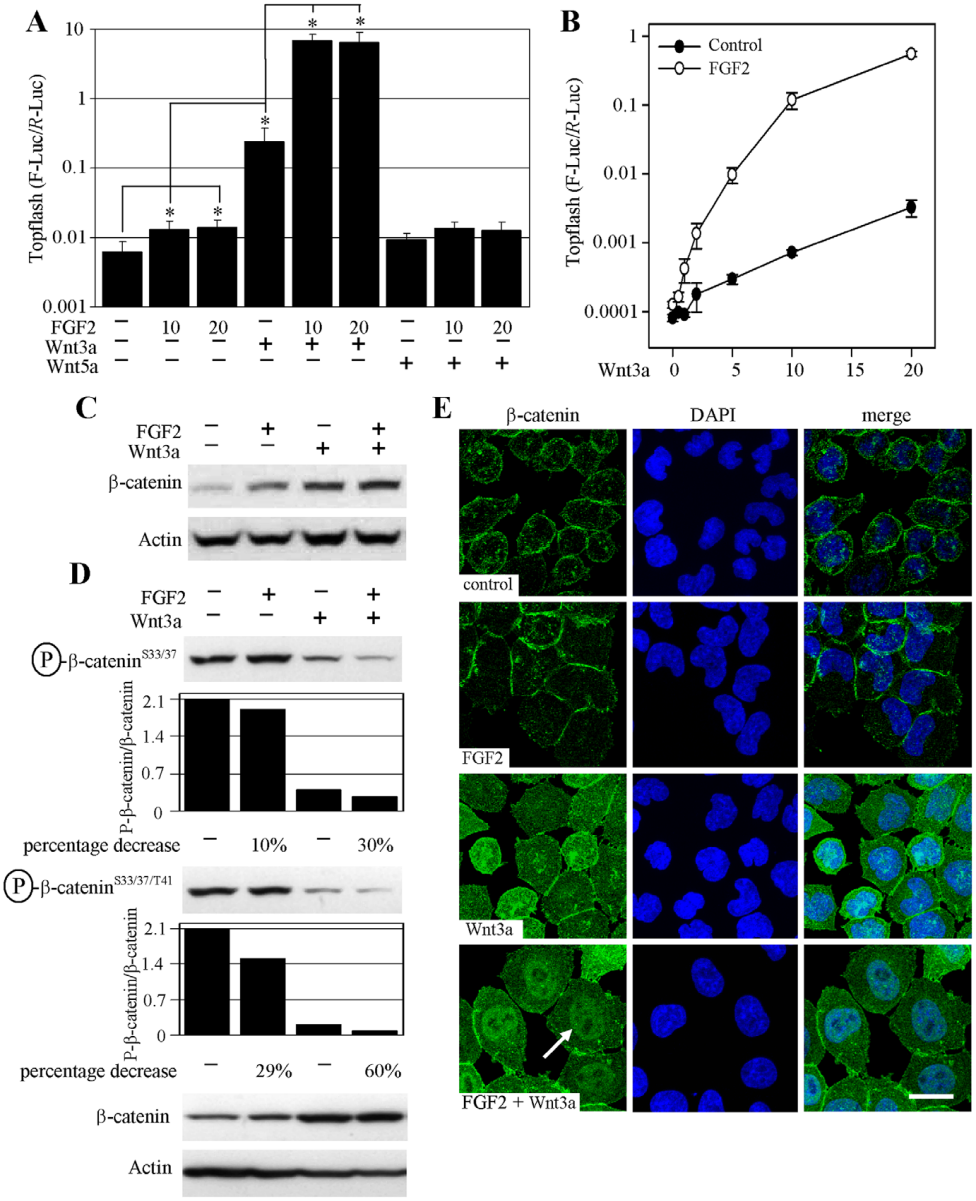
FGF2 activates WNT/β-catenin signaling. (A) RCS cells were transfected with Topflash firefly luciferase (F-Luc) and control *Renilla* luciferase (*R*-Luc) vectors, treated with FGF2 (ng/ml), WNT3a and WNT5a (40 ng/ml) and analyzed for luciferase activity 20 hours later. Data represent an average from 4 transfections (each measured twice), with the indicated standard deviations (* *p*<0.001; Student’s *t*-test; compared to control). Non-canonical WNT5a serves as a negative control. A logarithmic scale for the *y*-axis is necessary to show the massive Topflash activation induced by FGF2/WNT3a. Results are representative of 10 experiments. (B) Cells were transfected similar to (A), treated with FGF2 (20 ng/ml) and WNT3a (0.5–20 ng/ml) and analyzed for luciferase activity. Data represent an average from three transfections (each measured twice), with the indicated standard deviations. Results are representative of three experiments. (C) Cells were treated with FGF2 (20 ng/ml) and WNT3a for 1 hour and analyzed for total β-catenin by WB (signal quantified by densitometry). (D) Cells were treated as indicated for one hour and the levels of GSK3-mediated phosphorylation of β-catenin at Ser33/37 and Thr41 were monitored by appropriate antibodies. The WB signal was quantified by densitometry, normalized to total β-catenin and graphed. Note the FGF2 effect on β-catenin phosphorylation which is more profound in the presence of WNT3a (percentage decrease of signal in FGF2 alone treated cells vs. control, and FGF2/WNT3a treated cells vs. WNT3a treated cells). (E) Cells were treated with FGF2 and WNT3a for 2 hours and analyzed for β-catenin by direct immunocytochemistry. WNT3a induces cytoplasmic and nuclear β-catenin compared to membranous staining in control or FGF2-treated cells. Note the prominent nuclear staining in cells treated with FGF2/WNT3a (arrow). Bar 25 µm.

**Figure 2:**
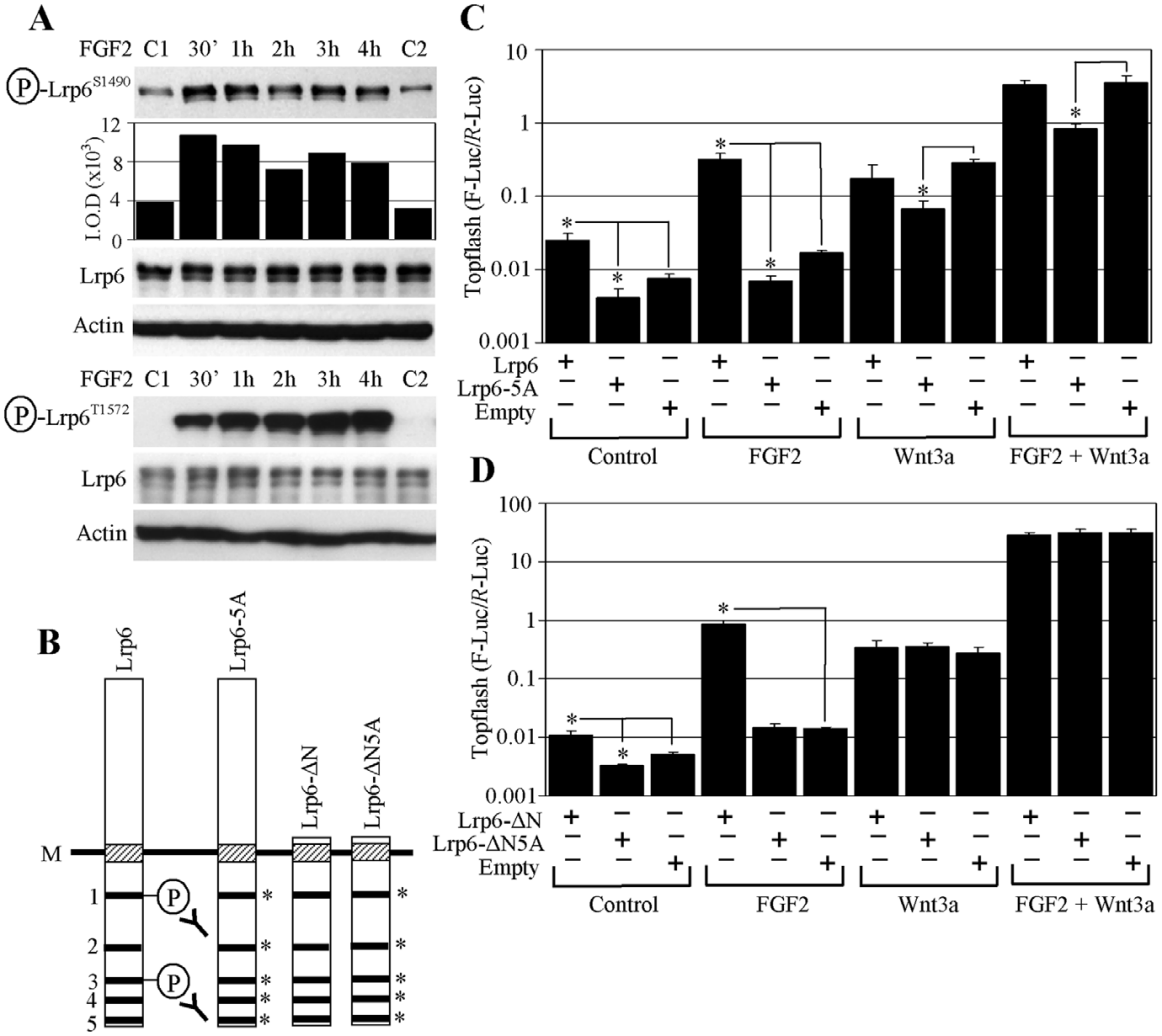
FGF2 activates WNT/β-catenin signaling via LRP6 phosphorylation. (A) FGF2-treated RCS cells were analyzed by WB for LRP6 phosphorylation at Ser1490 (signal quantified by densitometry) or Thr1572. Total LRP6 and ACTIN serve as loading controls. C1, C2 - untreated cells. (B) Schematic representation of the LRP6 expression vectors: intracellular PPPS/TP motifs are indicated (1–5), including the position of Ser1490 (motif 1) or Thr1572 (motif 3) recognized by the antibodies used in (A). M - cell membrane. Asterisks indicate Ser/Thr in the PPPS/TP motifs that were replaced by Ala. (C, D) Cells were transfected with LRP6 or empty vector together with Topflash reporter vectors, treated as indicated, and analyzed for luciferase activity. Data represent an average from four transfections (each measured twice), with the indicated standard deviations (* *p*<0.001; Student’s *t*-test). Results are representative of three experiments.

## Results

### Fibroblast Growth Factor (FGF) Signaling Activates WNT/β-catenin Signaling Independent of the PI3K/AKT Pathway

When probing rat chondrosarcoma chondrocytes (RCS) for effects of FGF signaling, we found FGF2-mediated upregulation of the Topflash luciferase reporter, which records the transcriptional activation of canonical WNT/β-catenin pathway. FGF2 activated Topflash in all 10 experiments conducted (129±74%; average±S.D. percentage of Topflash activity increase compared to untreated cells; 10 ng/ml FGF2; n = 10) (Fig. 1A). When combined, WNT3a and FGF2 caused a surprisingly potent Topflash activation, exceeding, in some cases, by more than 100 fold activation caused by WNT3a alone (Fig. 1A). This was confirmed by exposing cells to a range of WNT3a concentrations (0.5–20 ng/ml), in the presence of a single FGF2 dose. FGF2 potently enhanced WNT3a-mediated Topflash activation throughout the entire concentration range (Fig. 1B). The Topflash activation correlated with stabilization of β-catenin in cells treated with FGF2 and/or WNT3a, as detected by western blotting (WB) (Fig. 1C). We next determined the levels of GSK3-mediated β-catenin phosphorylation at Ser37/33 and Thr41, which takes place in the β-catenin destruction complex and targets β-catenin for proteasome-mediated degradation [1]. WNT3a decreased β-catenin phosphorylation at Ser33/37/Thr41, as expected by dissolution of the destruction complex (Fig. 1D). Importantly, this phenotype was significantly enhanced in cells co-treated with FGF2 and WNT3a, in contrast to FGF2 treatment alone that had only a weak influence over β-catenin phosphorylation. By direct β-catenin immunocytochemistry, both untreated cells and those treated with FGF2 showed mostly a membranous signal accumulated in areas of intercellular contact, likely representing β-catenin associated with membranous cadherins. WNT3a induced strong, equally cytoplasmic and nuclear β-catenin staining, due to stabilization of cytoplasmic β-catenin and its subsequent nuclear accumulation. Importantly, the nuclear β-catenin staining was significantly increased in cells treated with both WNT3a and FGF2, further confirming that the massive Topflash activation in cells treated with FGF2/WNT3a was caused by transcriptional activity of nuclear β-catenin (Fig. 1E). Collectively, our data demonstrate that although FGF2 alone is capable of Topflash induction, its main effect on WNT/β-catenin signaling lies in a potent sensitization of cells to exogenous WNT3a.

**Figure 3:**
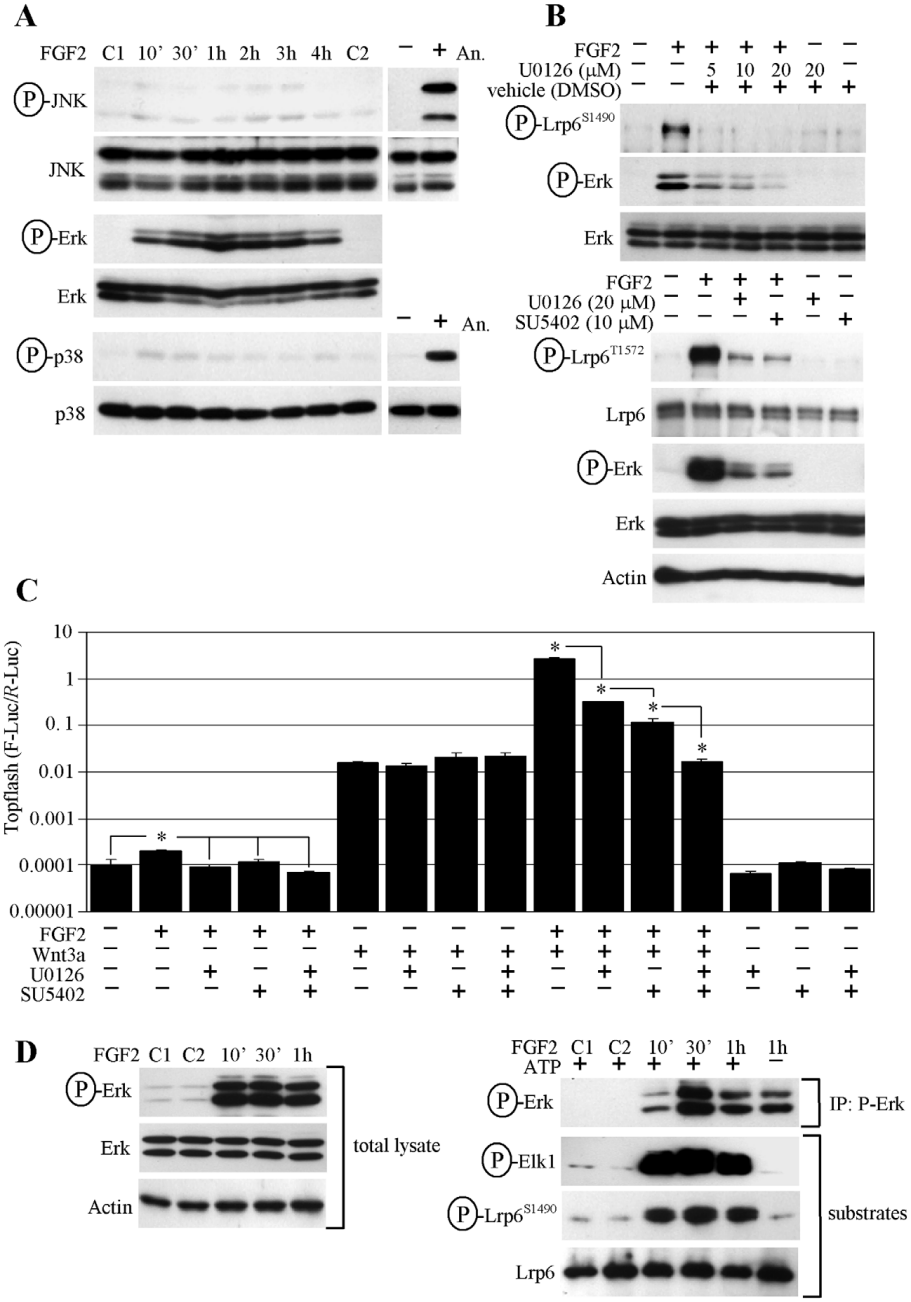
FGF2 uses ERK MAP kinase to phosphorylate LRP6. (A) RCS cells were treated as indicated and analyzed for activating phosphorylation of JNK, ERK and p38 MAP kinases by WB (C1, C2 - untreated controls). Anizomycin (An.; 10 µg/ml, 1.5 hour) serves as positive control for JNK and p38 activation. (B) Cells were treated with the MEK inhibitor U0126 or FGFR inhibitor SU5402 for 30 minutes prior to FGF2 treatment and analyzed for the indicated molecules. (C) Cells were transfected with Topflash reporter vectors, treated with the U0126 (15 µM) and FGFR inhibitor SU5402 (7 µM) for 1 hour prior to FGF2 and WNT3a addition, and analyzed for luciferase activity. A logarithmic scale for the *y*-axis is necessary to show the massive Topflash activation induced by FGF2/WNT3a. The data represent an average from three transfections (each measured twice), with the indicated standard deviations (* *p*<0.001; Student’s *t*-test). Results are representative of three experiments. (D) Active ERK was immunoprecipitated (IP) from cells treated with FGF2 (left panel), and subjected to a kinase assay with either recombinant ELK1 or LRP6 as a substrate (right panel). A sample with ATP omitted serves as a negative control.

**Figure 4:**
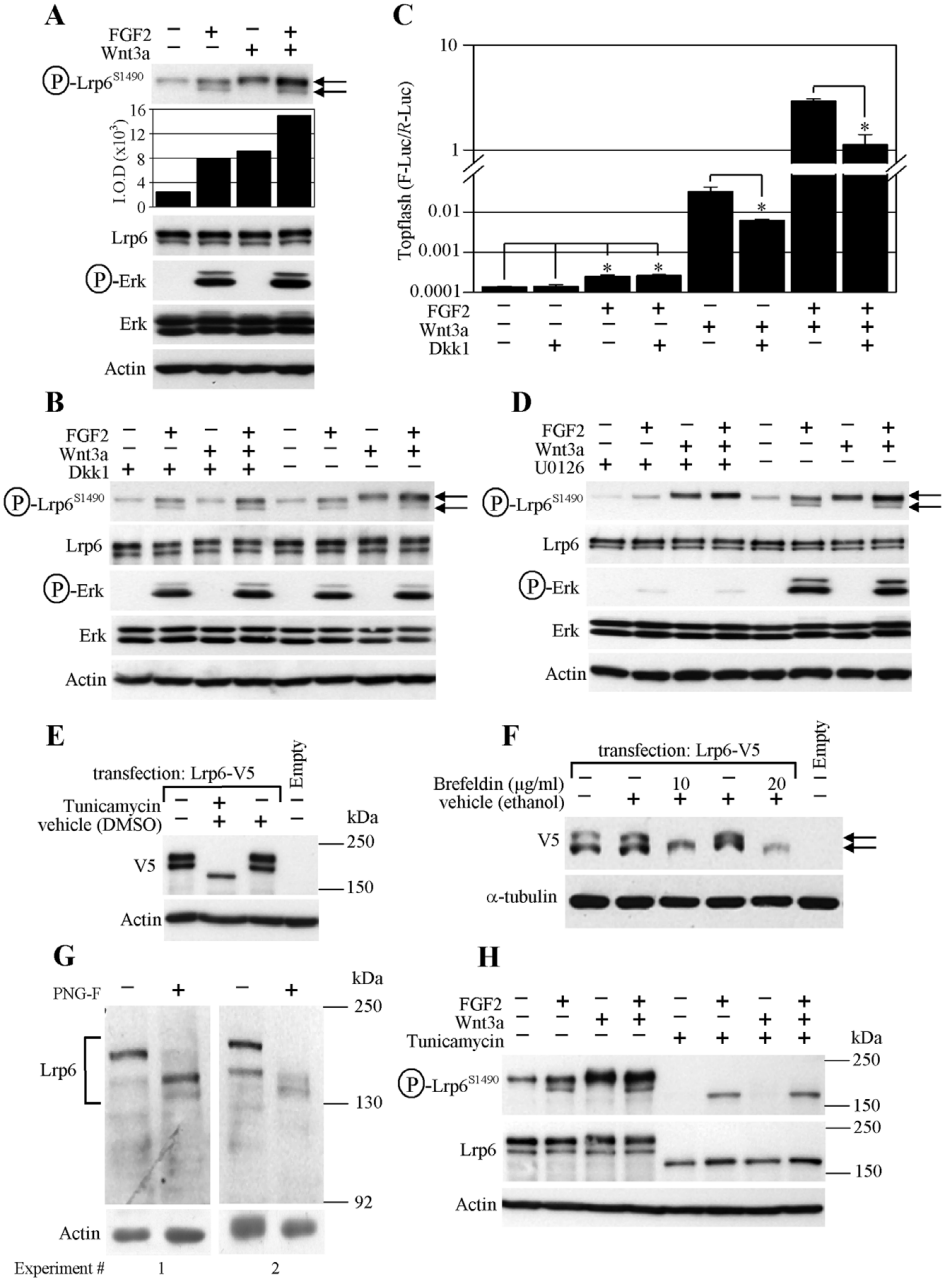
ERK phosphorylates LRP6 along its Golgi-based maturation pathway. (A) The LRP6 phosphorylation induced in RCS cells by FGF2 and WNT3a was determined by WB and quantified by densitometry. LRP6 migrates as two bands, differently phosphorylated in cells treated by FGF2 and WNT3a (arrows). (B, C) Addition of recombinant DICKKOPF1 (Dkk1) prevents WNT3a-mediated LRP6 phosphorylation (B; upper LRP6 band) and Topflash activation (C), but not that induced by FGF2. (D) Inhibition of ERK pathway by U0126 (10 µM) prevents FGF2-mediated LRP6 phosphorylation (both bands; arrows) while it does not affect WNT3a-mediated phosphorylation (upper band). (E) RCS cells were transfected with V5-tagged LRP6 or empty plasmid, incubated in the presence of tunicamycin (0.1 µM) for 24 hours, and analyzed for indicated molecules by WB. (F) HEK293 cells were transfected with V5-tagged LRP6 plasmid and treated with brefeldin A for 24 hours. Empty vector (pcDNA3) serves as a control for transfection; ACTIN or α-TUBULIN serve as loading controls. LRP6 fails to fully mature in cells treated with tunicamycin or brefeldin A (arrows), which inhibit glycosylation or protein transport from the endoplasmatic reticulum to the Golgi network, respectively. (G) A whole RCS cell lysate was incubated with N-glycosidase F (PNG-F) for 16 hours and analyzed for LRP6 by WB. (H) RCS cells were cultivated with tunicamycin (5 µM) for 24 hours prior to FGF2 and WNT3a (1 hour) treatment. Note the lack of WNT3a-mediated LRP6 phosphorylation when only the immature, non-glycosylated LRP6 is produced as a result of tunicamycin treatment.

We next asked whether the PI3K/AKT pathway accounts for the effect of FGF2 on WNT3a/β-catenin signaling. FGF2 induced some PI3K/AKT activation in RCS cells, as determined by WB for AKT phosphorylation at Ser473 or a cell-free AKT kinase assay. AKT activation was accompanied by increased inhibitory phosphorylation of GSK3α at Ser21 (Fig. S1A, B). However, chemical inhibition of PI3K by wortmannin or LY294002 did not significantly alter the effect of FGF2 and WNT3a on Topflash activation (Fig. S1C). Similarly, overexpression of constitutively-active AKT led to increased phosphorylation of GSK3α at Ser21 but showed no effect on FGF2 and/or WNT3a-mediated activation of Topflash (Fig. S1D, E).

**Figure 5:**
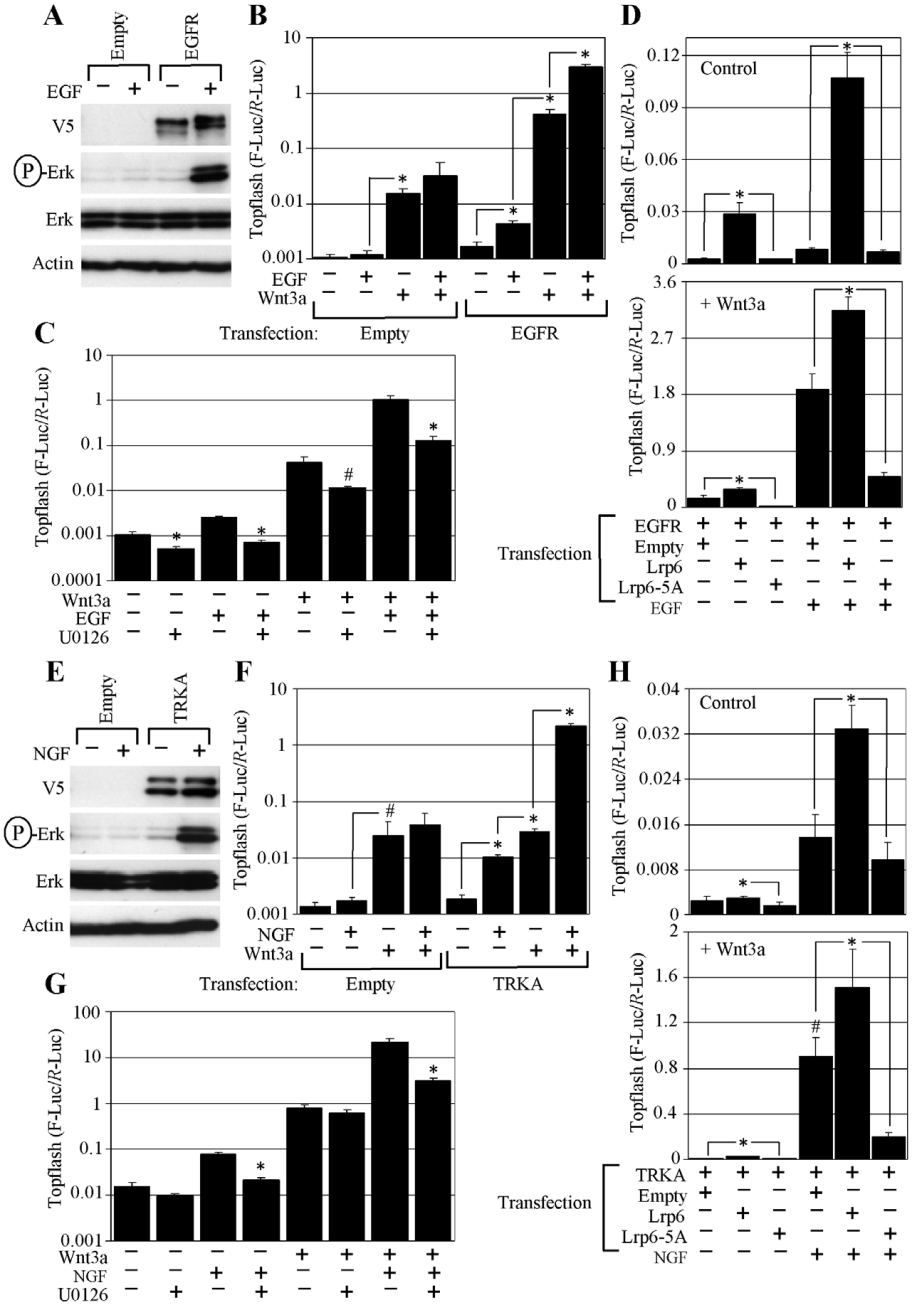
EGFR and TRKA activate WNT/β-catenin signaling via ERK/LRP6 pathway. (A, E) RCS cells were transfected with empty plasmid or plasmid encoding V5-tagged EGFR or TRKA, treated with EGF or NGF (50 ng/ml) for 1 hour, and analyzed for indicated molecules by WB. (B, F) Cells were transfected as indicated, grown for 24 hours, treated with EGF, NGF and WNT3a, and analyzed for luciferase activity. Data represent an average from three transfections (each measured twice). Statistically significant differences are indicated (* *p*<0.0001, # *p*<0.05; Student’s *t*-test). Note the potent upregulation of basal or WNT3a-mediated Topflash activity in EGF or NGF-treated cells expressing the corresponding receptor. (C, G) Cells were transfected with EGFR (C) or TRKA (G) together with Topflash reporter vectors, treated with U0126 (20 µM) one hour prior to EGF, NGF and WNT3a treatment, and analyzed for luciferase activity. Data represent an average from three or four transfections (each measured twice). Statistically significant differences are indicated (* *p*<0.0001, # *p*<0.005; Student’s *t*-test, compared to cells without U0126 for each treatment). (D, H) Cells were transfected as indicated, treated with EGF, NGF and WNT3a for 48 hours, and analyzed for luciferase activity. Data represent an average from four transfections (each measured twice), with the indicated standard deviations. Statistically significant differences are indicated (* *p*<0.0001, # *p*<0.001; Student’s *t*-test).

### FGF2 Induces Phosphorylation of the WNT3a co-receptor LRP6

Examining the WNT/β-catenin pathway for components that were subject to FGF2-induced phosphorylation revealed WNT co-receptor LRP6 phosphorylation at Ser1490 and Thr1572 (Fig. 2A). Ser1490 and Thr1572 lie within the two of the five conserved PPPS/TP motifs present in the intracellular domain of LRP6. To test whether the FGF2 effect on WNT/β-catenin signaling depended upon phosphorylation within the PPPS/TP motifs, we transfected RCS cells with a LRP6 mutant with its Ser/Thr residues in each particular PPPS/TP motif replaced by Ala (LRP6-5A) (Fig. 2B, C). LRP6-5A showed a dominant-negative effect on both FGF2 and/or WNT3a-mediated Topflash activation, contrasting with overexpression of wild-type (wt) LRP6, which led to spontaneous phosphorylation at Ser1490 and significantly enhanced FGF2-mediated Topflash activation (Figs S2A, 2C). Similar results were achieved with a truncated LRP6 variant, which lacks the entire extracellular domain of LRP6 (LRP6- ΔN). As expected, LRP6- ΔN did not interfere with exogenous WNT3a-mediated activation of Topflash, but markedly enhanced activation by FGF2 (Fig. 2D).

**Figure 6:**
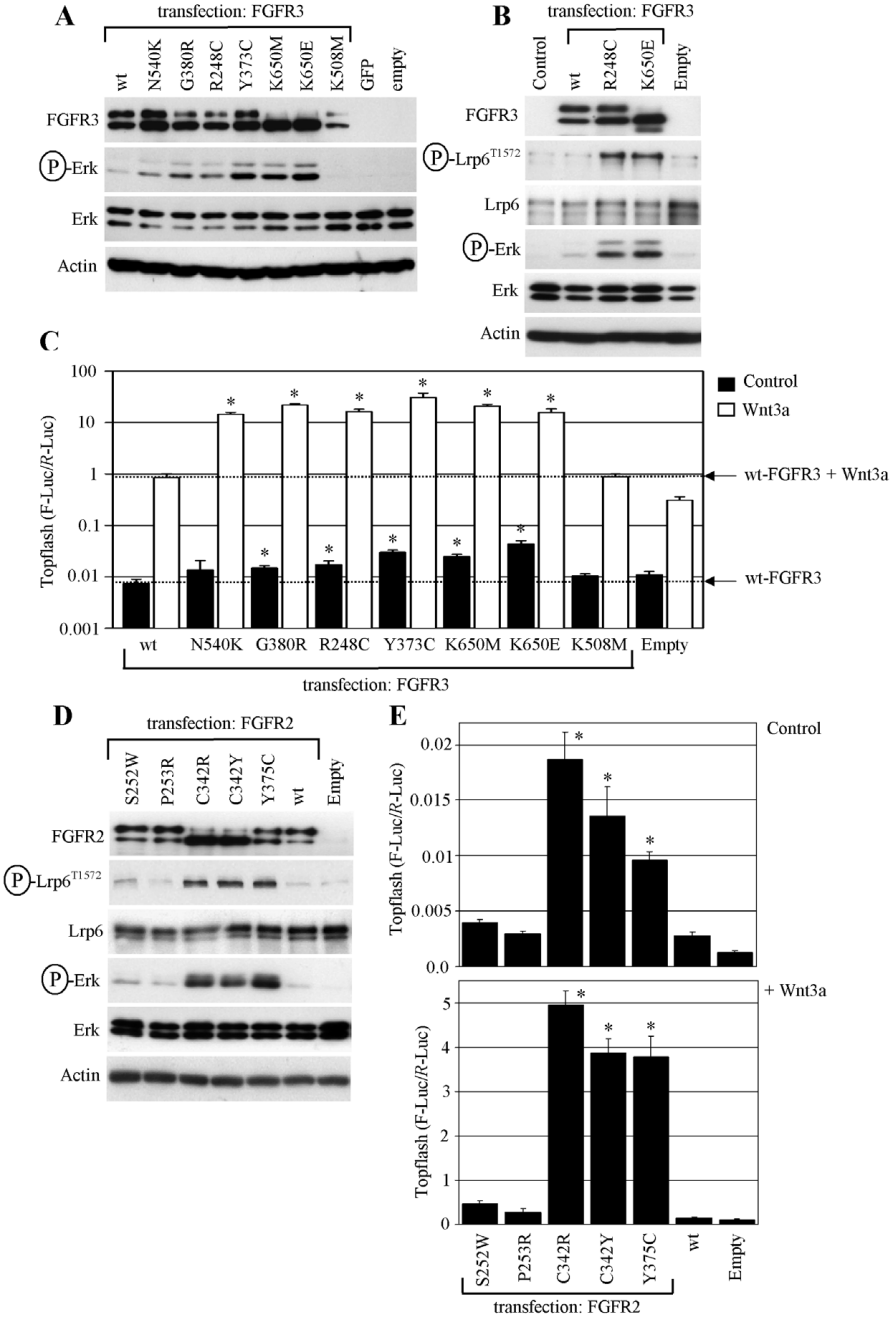
Disease-associated FGFR3 and FGFR2 mutants signal via ERK/LRP6 pathway. (A) RCS cells were transfected with wt FGFR3 or activating FGFR3 mutants (N540K, G380R, R248C, Y373C, K650M, K650E), and analyzed for the indicated molecules by WB 48 hours later. The levels of ERK phosphorylation vary among the tested mutants, reflecting the different strength of FGFR3 activation by each particular mutation [24]. K508M - kinase inactive FGFR3 mutant. GFP and empty vectors serve as transfection controls. (B) LRP6 phosphorylation at Thr1572 caused by highly activating FGFR3 mutants R248C and K650E. (C) Cells were transfected with the indicated FGFR3 vectors together with Topflash reporter vectors, treated with WNT3a and analyzed for luciferase activity. Data represent an average from three transfections (each measured twice), with the indicated standard deviations. A logarithmic scale of the *y*-axis is necessary to express the massive Topflash activation in WNT3a-treated cells expressing activating FGFR3 mutants (* *p*<0.001; Student’s *t*-test; compared to wt FGFR3). Results are representative of four experiments. (D) Cells were transfected with wt FGFR2 or activating FGFR2 mutants (S252W, P253R, C342R, C342Y, Y375C), and analyzed for the indicated molecules by WB. Note the significant ERK and LRP6 phosphorylation caused by C342R, C342Y and Y375C mutants, which correlates with increased basal (E; upper graph) and WNT3a-induced (E; lower graph) β-catenin activity, evidenced by Topflash experiment. Results are representative for three experiments (* *p*<0.001; Student’s *t*-test; compared to wt FGFR2).

### FGF Signaling Employs ERK MAP Kinase to Phosphorylate LRP6

FGF2 effect on the WNT3a/β-catenin pathway requires LRP6 phosphorylation at its PPPS/TP motifs (Fig. 2). Recently, we showed that all three MAP kinases, i.e. JNK, p38 and ERK, are capable of phosphorylating the PPPS/TP motifs in LRP6 [8]. In RCS cells, only ERK was strongly activated by FGF2 (Fig. 3A), and chemical inhibition of MEK (a kinase upstream of ERK) by U0126 or FGFR3 by SU5402 suppressed FGF2-mediated LRP6 phosphorylation (Fig. 3B). Similarly, FGF2 but not WNT3a-mediated Topflash activation was sensitive to U0126, SU5402 or a combination of both drugs (Fig. 3C). To further confirm that ERK acts as an LRP6 kinase in RCS cells, we immunoprecipitated active ERK from FGF2-treated RCS cells, and subjected the immunocomplexes to a kinase assay with recombinant LRP6 as a substrate. We show ERK-mediated LRP6 phosphorylation at Ser1490 in a kinase assay (Fig. 3D). Since the Ser1490 antibody is designed to detect phosphorylation only at the first PPPS/TP motif, we used mass-spectrometry (MS) to probe LRP6 for ERK-mediated phosphorylation at the remaining four motifs. We identified phosphorylation on the first four N-terminal PPPS/TP motifs, but were not able to detect the peptides containing the most C-terminal PPPS/TP motif within the MS spectras (data not shown).

**Figure 7:**
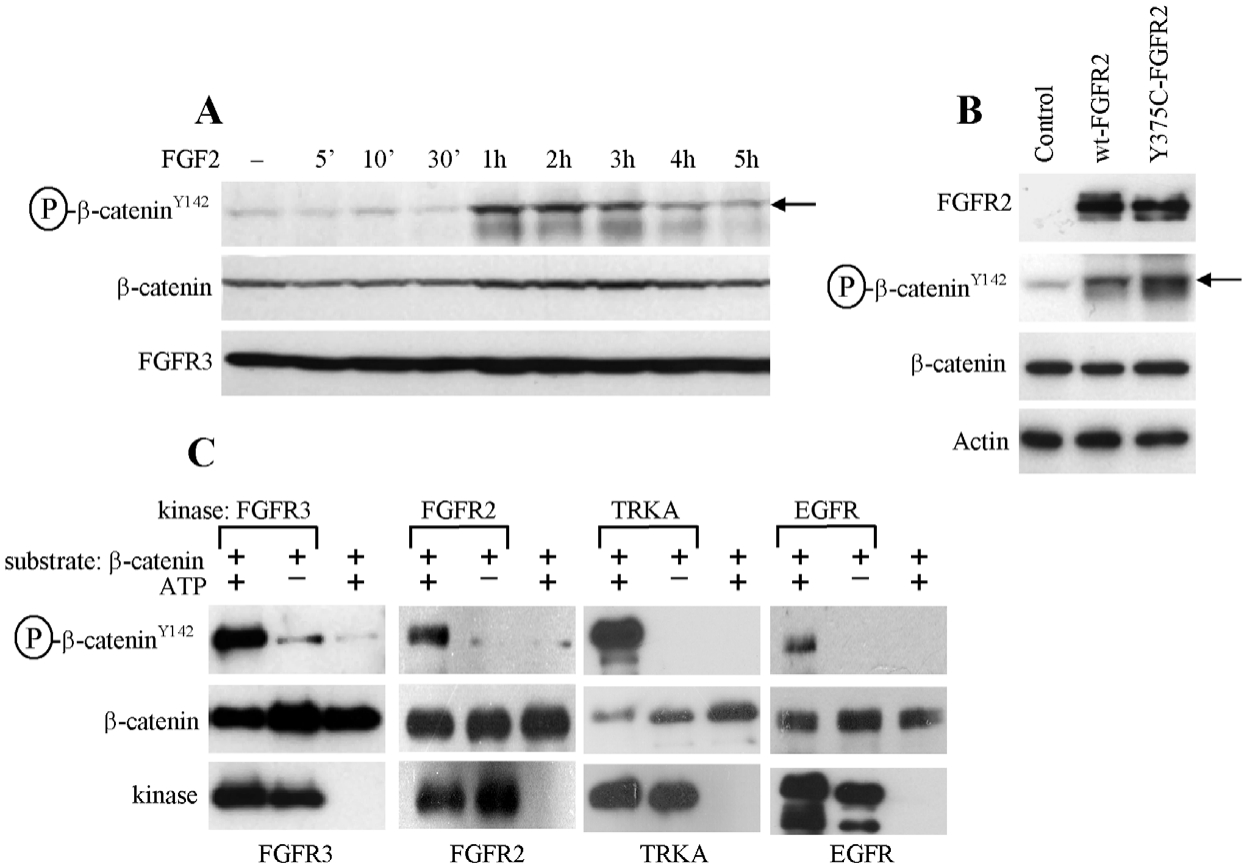
RTKs phosphorylate β-catenin at Tyr142. (A) RCS cells were treated for indicated times with FGF2 (10 ng/ml) in the presence of heparin (1 µg/ml), and analyzed for β-catenin phosphorylation at Tyr142 by WB (arrow). (B) HEK293 cells were transfected with wt FGFR2 or its activating mutant Y375C, and analyzed for indicated molecules 48 hours later. Note the increased β-catenin phosphorylation at Tyr142 (arrow). (C) Active recombinant FGFR3, FGFR2, TRKA and EGFR were subjected to a cell-free kinase assay with recombinant β-catenin as a substrate. Samples with ATP or kinase omitted serve as controls for kinase reaction.

### ERK MAP Kinase Phosphorylates LRP6 along its Golgi-based Maturation Pathway

Detailed analysis of the endogenous LRP6 SDS-PAGE migration pattern shows two discrete protein bands in RCS cells. Interestingly, while treatment with FGF2 caused phosphorylation of both the upper and lower LRP6 bands, WNT3a only induced phosphorylation of the upper form (Fig. 4A). WNT3a-mediated phosphorylation of the upper LRP6 band decreased in the presence of exogenously added recombinant DICKKOPF1 (DKK1), which inhibits interaction of WNT3a with the FRIZZLED/LRP6 receptor complex [9]. In contrast, FGF2-mediated phosphorylation of both the upper and lower LRP6 bands was unaffected by DKK1. Similarly, DKK1 inhibited WNT3a but not FGF2-mediated activation of Topflash (Fig. 4B, C). Treatment with U0126 produced an effect opposite to DKK1, inhibiting FGF2-mediated phosphorylation of both LRP6 bands, while leaving WNT3a-mediated phosphorylation of the upper LRP6 band intact (Fig. 4D). These data correspond to the U0126 effect in the Topflash experiment (Fig. 3C).

**Figure 8:**
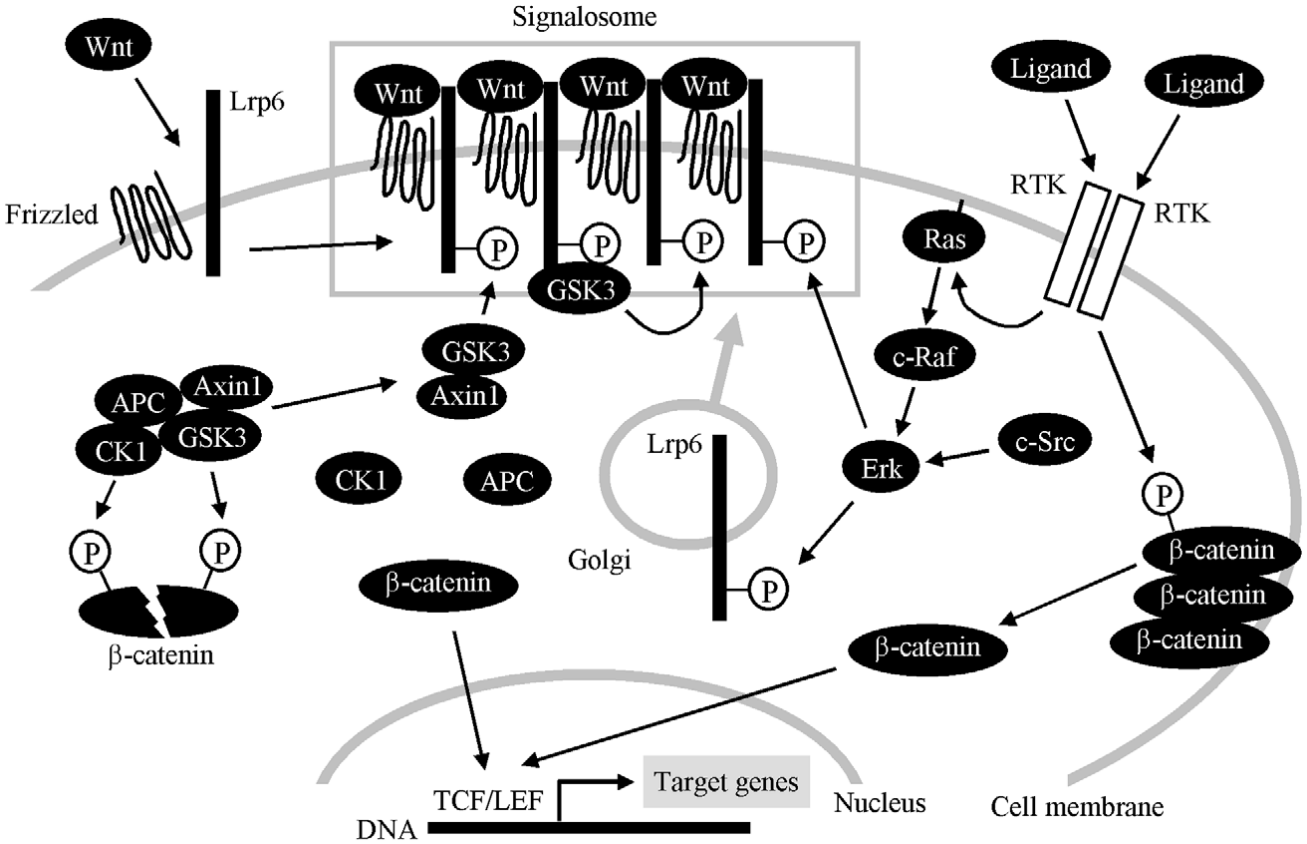
A proposed model of RTK and WNT/β-catenin signaling cross-talk. In the basal cell state, cytoplasmic β-catenin levels are low due to rapid turnover mediated by the destruction complex (AXIN1, APC, CK1 and GSK3), where CK1 and GSK3-mediated phosphorylation targets β-catenin for degradation. WNT binds to its cell surface receptors FRIZZLED and LRP6, inducing the clustering of WNT/FRIZZLED/LRP6 into the multimeric complexes called signalosomes. In signalosomes, LRP6 becomes phosphorylated at PPPS/TP motifs, which allows for AXIN1 and GSK3 binding. Signalosomes also facilitate the amplification of the WNT signal, where initially phosphorylated LRP6 molecules serve as high affinity docking sites for GSK3 that, in turn, phosphorylates additional LRP6 molecules to create even more AXIN1/GSK3 binding sites. AXIN1/GSK3 sequestration by LRP6 leads to dissolution of the destruction complex, allowing for β-catenin stabilization, its nuclear translocation, and activation of gene transcription dependent on TCF/LEF transcription factors. WNT-induced LRP6 phosphorylation requires signalosome assembly and therefore can only involve the mature, transmembrane LRP6. This contrasts with the ERK-mediated LRP6 phosphorylation, since ERK, activated by RTKs, is a cytosolic kinase than can phosphorylate both the mature (transmembrane) LRP6 and immature (intracellular) LRP6 during its Golgi-based membrane transport (gray arrow). In the absence of a signalosome, ERK-phosphorylated LRP6 may recruit a limited amount of AXIN1/GSK3 that is not sufficient to fully stabilize β-catenin. In the presence of WNT, however, LRP6 molecules pre-phosphorylated by ERK integrate into the newly formed signalosomes and help to amplify the WNT signal by providing more initial binding sites for AXIN1/GSK3. In addition to the ERK/LRP6 pathway, RTKs also directly phosphorylate β-catenin at Tyr142, possibly liberating β-catenin from its association with the cell membrane, and allowing for its transcriptional activation.

Transfected LRP6 also migrated as two bands when expressed in RCS or HEK293 cells. Treatment with the glycosylation-inhibitor tunicamycin, or the Golgi-transport inhibitor brefeldin resulted in disappearance of the upper LRP6 band (Fig. 4E, F). De-glycosylation of a RCS whole cell lysate produced a similar LRP6 migration pattern (Fig. 4G), together suggesting that the upper LRP6 band represents the mature, glycosylated form present at the cell surface, whereas the lower band is an immature LRP6, undergoing glycosylation during its Golgi-mediated transport to the cell surface. Consistent with this hypothesis, WNT3a failed to induce LRP6 phosphorylation in RCS cells devoid of the upper (membranous) LRP6 band due to tunicamycin treatment, while FGF2-mediated phosphorylation of the lower (intracellular) LRP6 band was preserved (Fig. 4H).

### RTKs Activate WNT/β-catenin Signaling via ERK/LRP6 Pathway

The ERK MAP kinase represents a common signaling intermediate utilized by many systems. We therefore asked whether the signaling systems unrelated to FGF are also capable to signal via the ERK/LRP6 pathway. First, we focused on small GTPase RAS and RAF-1 kinase that both lie upstream of ERK in the ERK signaling module. Expression of constitutively active forms of RAS (RasV12) or RAF-1 (RafCAAX) lead to ERK activation, and corresponding strong induction of basal or WNT3a-mediated Topflash activity which was sensitive to U0126 (Fig. S3A, B). Moreover, RasV12 or RafCAAX-mediated activation of WNT/β-catenin signaling was significantly enhanced in cells co-expressing wt LRP6, whereas it was inhibited via dominant-negative LRP6 mutant LRP6-5A (Fig. S3C).

We next asked whether other signaling systems signal utilize ERK/LRP6 pathway. Expression of constitutively active variant of non-receptor tyrosine kinase SRC (SRC-Y529F) in RCS cells resulted in ERK activation accompanied by potent induction of basal or WNT3a-mediated Topflash activity, which was inhibited by U0126 (Fig. S3D, E). Moreover, co-expression of wt LRP6 with SRC-Y529F enhanced the SRC-mediated stimulatory effect on Topflash activation, whereas expression of dominant-negative LRP6-5A mutant suppressed the latter (Fig. S3F). Next, we expressed 2 additional RTKs, EGFR and TRKA in RCS cells, and analyzed their effect on basal or WNT3a-mediated Topflash induction. Both EGFR and TRKA induced ERK activation in RCS cells and upregulated the WNT3a-mediated Topflash induction (Fig. 5A, B, E, F). EGFR and TRKA effect on WNT3a-mediated Topflash induction was abolished by U0126 or expression of dominant-negative LRP6-5A mutant (Fig. 5C, D, G, H), demonstrating that both EGFR and TRKA signal via ERK/LRP6 pathway to upregulate WNT/β-catenin signaling.

### Human Disease-associated FGFR2 and FGFR3 Mutants Signal via ERK/LRP6 Pathway

Among the FGFRs, RCS cells express FGFR2 and FGFR3, and we showed the effect of endogenous, wt FGFR2 and FGFR3 activation on WNT/β-catenin signaling (Fig. 1). To test whether the WNT/β-catenin activation occurs with FGFR2 and FGFR3 harboring pathogenic mutations, we used 6 activating FGFR3 mutants (N540K, G380R, R248C, Y373C, K650M, K650E), and 5 activating FGFR2 mutants (S252W, P253R, C342R, C342Y, Y375C), known to be associated with human skeletal dysplasias, cranial malformations, skin, and cancer [10], [11]. Ectopic expression of the FGFR3 mutants results in their activation via spontaneous dimerization, with the activating potential of the given mutation easily appreciated by the level of ERK phosphorylation (Fig. 6A) [12]. We also demonstrate the Thr1572 phosphorylation of LRP6, which took place in cells expressing highly activating FGFR3 mutants R248C and K650E (Fig. 6B). Topflash activation by FGFR3 mutants significantly exceeded that of wt FGFR3 (Fig. S4), and this induction was increased by expression of wt LRP6, or abolished by the dominant-negative LRP6-5A mutant (Fig. S2B, C).

Among the FGFR2 mutants, expression of C342R, C342Y and Y375C FGFR2 resulted in spontaneous ERK activation and corresponding LRP6 phosphorylation at Thr1572 (Fig. 6D). Moreover, experiments using cells treated with WNT3a demonstrated that FGFR3 and ERK-activating FGFR2 mutants also potently sensitize cells to WNT3a (Fig. 6C, E). Similar results were obtained in PC12 cells overexpressing activating FGFR3 mutant K650E (Fig. S5).

### FGFR2, FGFR3, EGFR and TRKA Phosphorylate β-catenin at Tyr142

When used alone, FGF2 increased basal Topflash levels in RCS cells (Figs 1A, B; 2C, D; 3C). These results appeared to be unrelated to the FGF2 effect on autocrine WNT signaling since it was insensitive to the exogenously added DKK1 (Fig. 4C, cells treated with FGF2 alone), suggesting an alternative mechanism. In RCS cells, FGF2 treatment led to β-catenin phosphorylation at Tyr142 (Fig. 7A) as did overexpression of wt FGFR2 or activating FGFR2 mutant Y375C in HEK293 cells (Fig. 7B). Recombinant FGFR3 and FGFR2 caused β-catenin Y142 phosphorylation in a cell-free kinase assays utilizing recombinant β-catenin as a substrate. Similar β-catenin phosphorylation was found in the kinase assays utilizing recombinant EGFR and TRKA as kinases (Fig. 7C). This correlated with the Topflash experiment, where treatment with EGF or NGF caused Topflash activation in RCS cells overexpressing EGFR or TRKA, respectively (Fig. 5B, F; cells treated with EGF or NGF alone).

## Discussion

A critical event in WNT/β-catenin signaling appears to be the phosphorylation within the intracellular PPPS/TP motifs of LRP6. Removal of any of the five PPPS/TP motifs impairs WNT signaling; removal of all five motifs results in complete loss of WNT/β-catenin signal transduction [13], [14]. Given their importance, the PPPS/TP motifs represent a major site for modulation of the WNT/β-catenin pathway by other signaling systems. However, the nature of the kinases and phosphatases targeting the PPPS/TP motifs is only beginning to emerge [15]. Here, we found that RTK signaling mediated by FGFR2, FGFR3, TRKA and EGFR kinases activates WNT/β-catenin signaling via a mechanism which involves phosphorylation within the PPPS/TP motifs of LRP6. We further demonstrate that RTKs employ ERK MAP kinase to activate WNT/β-catenin signaling via LRP6 phosphorylation.

How does the RTK/ERK-mediated LRP6 phosphorylation activate WNT/β-catenin signaling? Activation of FGFR2 and FGFR3, via cell treatment with FGF2 ligand, induced total LRP6 phosphorylation at levels similar to WNT3a (Fig. 4A), yet it alone produced only small increase in Topflash activity compared to WNT3a and especially compared to combined FGF2/WNT3a (Fig. 1A). These data suggest that FGF2-mediated phosphorylation of LRP6 is not sufficient to significantly stabilize β-catenin without a concomitant WNT3a signal, but instead potently enhances β-catenin signaling in the presence of WNT3a. Phosphorylation within the PPPS/TP motifs of LRP6 plays a critical role in WNT/β-catenin signaling by providing docking sites to bind AXIN1 and GSK3, thereby sequestering both proteins away from the β-catenin destruction complex [13], [14]. However, the precise chronology of events is somewhat unclear because both AXIN1 and GSK3 bind LRP6 only when phosphorylated at PPPS/TP motifs, but GSK3 also serves as a canonical PPPS/TP kinase in the WNT/β-catenin pathway [13], [14], [16], [17]. Although isolated LRP6 molecules can bind GSK3 and AXIN1, this appears insufficient to propagate the WNT/β-catenin signal in cells, where WNT/FRIZZLED/LRP6 complexes must aggregate into large, ribosome-sized complexes called signalosomes built around the multimeric scaffolding protein DISHEVELLED [18]. Concentrated in signalosomes, LRP6 displays higher avidity for AXIN1/GSK3. More importantly, signalosomes facilitate the amplification of the WNT signal, since initially phosphorylated LRP6 molecules serve as high affinity docking sites for AXIN1/GSK3 that, in turn, phosphorylate additional LRP6 molecules to create even more binding sites for AXIN1 and GSK3 [15].

WNT-induced LRP6 phosphorylation requires signalosome assembly and thus can only target the fully mature, transmembrane form of LRP6. This differs from ERK-mediated LRP6 phosphorylation, since ERK is a cytosolic kinase that can phosphorylate both the mature (transmembrane) LRP6 and immature (intracellular) LRP6 during its Golgi-based cell membrane translocation/maturation pathway (Fig. 4). We speculate that, in the absence of signalosomes, ERK-mediated LRP6 phosphorylation may induce limited AXIN1/GSK3 recruitment and is not sufficient to fully stabilize β-catenin. In the presence of WNT, however, the LRP6 molecules pre-phosphorylated by ERK integrate into the newly formed signalosomes and help amplify the WNT signal by providing more initial binding sites for AXIN1/GSK3 (Fig. 8).

When used alone, FGF2 increased basal Topflash levels in RCS cells. Although this induction appeared weak compared to Topflash activation caused by WNT3a or FGF2/WNT3a, it represented signal increase by more than 100% (Fig. 1A), when compared to basal Topflash levels. Interestingly, a similar Topflash induction was found in EGF or NGF-treated cells expressing EGFR or TRKA, respectively. It is unlikely that, at least in case of FGF2, this induction stems from FGF2-mediated enhancing effect on autocrine WNT activity, since addition of recombinant DKK1 did not affect FGF2-mediated Topflash induction, in contrast to WNT3a induced Topflash, that was reversed by recombinant DKK1. Interestingly, we found that activation of FGFR signaling in cells, via stimulation with FGF ligand or overexpression of FGFR receptor, leads to β-catenin phosphorylation at Tyr142. Moreover, FGFR3, FGFR2, TRKA and EGFR appear to act as β-catenin kinases since they are capable of direct β-catenin Tyr142 phosphorylation in a cell-free kinase assay. Tyr142 phosphorylation reduces the affinity of β-catenin for cadherin, disrupts β-catenin interaction with α-catenin, and promotes β-catenin association with BCL9-2 protein, altogether resulting in release of membrane-associated β-catenin into the cytoplasm, its nuclear translocation and increased transcription of WNT/β-catenin target genes [21]–[23]. Thus the RTK-mediated β-catenin phosphorylation at Tyr142 may provide an alternative way of activation of canonical WNT/β-catenin signaling, which is independent of ERK/LRP6 pathway (Fig. 8).

Our data predict that activation of WNT/β-catenin signaling via LRP6 phosphorylation may represent a common signaling theme since many diverse signaling systems utilize ERK MAP kinase. Moreover, we show that activating mutants of FGFR2 and FGFR3 signal via ERK/LRP6 pathway. These mutants are associated with more than 10 human conditions, including syndromes affecting skeleton (achondroplasia, thanatophoric dysplasia, Crouzon and Beare-Stevenson syndrome), skin (seborrhaeic keratosis, acanthosis nigricans), and cancer development (multiple myeloma, seminoma, endometrial and breast cancer) [10], [19], [20]. Our data open an intriguing question of the role of ERK/LRP6 pathway in diseases associated with FGFR2 and FGFR3 mutations. Experiments are now ongoing to address this possibility.

## Materials and Methods

### Cell Culture and De-glycosylation

RCS and HEK293 cells were propagated in DMEM media, supplemented with 10% FBS and antibiotics (Invitrogene, Carlsbad, CA). The growth factors and chemicals were obtained from the following manufacturers: FGF1, FGF2, WNT3a, WNT5a, DKK1, EGF, NGF (R&D Systems, Minneapolis, MN); heparin (Invitrogene); wortmannin, LY294002, U0126 (Cell Signaling, Beverly, MA); tunicamycin (Enzo Life Sciences, Plymouth, PA); anisomycin (Calbiochem, San Diego, CA); SU5402, brefeldin A (Tocris Bioscience, Ellisville, MO). For de-glycosylation, cells were lysed in 0.5% NP40 lysis buffer supplemented with protease inhibitors (Roche, Indianopolis, IN), and 25 µl of lysate was incubated with 3 µl of N-Glycosidase F (Roche) for 16 hours at 37°C.

### Western Blotting, Immunoprecipitation, Kinase Assays and Mass Spectrometry

Cells were lysed in buffer containing 50 mM Tris-HCl pH 7.4, 150 mM NaCl, 0.5% NP-40, 1 mM EDTA, 25 mM NaF, supplemented with proteinase inhibitors and 10 mM Na_3_VO_4_. Protein samples were resolved by SDS-PAGE, transferred onto a PVDF membrane and visualized by chemiluminiscence (Thermo Scientific, Rockford, IL). Integrated optical density (I.O.D.) of the WB signal was quantified by Scion Image software (Scion Corporation, Frederick, MA). The following antibodies were used: β-catenin (BD Biosciences, Rockville, MD); P-LRP6^T1572^ (Millipore, Billerica, MA); ERK1/2, P-ERK1/2^T202/Y204^, JNK, P-JNK^T183/Y185^, p38, P-p38^T180/Y182^, P-ELK1^S383^, AKT, P-AKT^S473^, GFP, GSK3, P-GSK3α^S21^, P-GSK3α/β^S21/9^, LRP6, P-LRP6^S1490^, P-β-catenin^S33/37^, P-β-catenin^S33/37/T41^ (Cell Signaling, Beverly, MA); P-β-catenin^Y142^ (ECM Biosciences, Versailles, KY); ACTIN, FGFR2, FGFR3, TRKA, EGFR (Santa Cruz Biotechnology, Santa Cruz, CA); V5 (Invitrogene); α-tubulin (Developmental Studies Hybridoma Bank, IA). AKT and ERK immunoprecipitations and kinase assays were performed according to the manufacturer’s protocol (Cell Signaling). For the recombinant ERK kinase assay, the reactions were carried-out with 300 ng of recombinant ERK and 600 ng of recombinant LRP6 in 50 µl of kinase buffer (60 mM HEPES-NaOH pH 7.5, 3 mM MgCl_2_, 3 mM MnCl_2_, 3 µM Na_3_VO_4_, 1.2 mM DTT) in the presence of 200 µM ATP for 30 minutes at 30°C. Recombinant LRP6 was described earlier [14]. For the recombinant RTK kinase assays, the reactions were carried-out with 400 ng of recombinant FGFR2, FGFR3, TRKA or EGFR (SignalChem, Richmond, Canada) and 400 ng of recombinant β-catenin (Abcam, Cambridge, MA, USA) in 50 µl of kinase buffer in the presence of 50 µM ATP for 60 minutes at 30°C. For mass spectrometry, protein bands were cut from SDS-PAGE gels and subjected to in-gel trypsin digestion. Electrospray MS was performed on the tryptic peptides using an LCQ Deca XP ion-trap mass spectrometer equipped with in-line liquid chromatography (Thermo Finnigan, West Palm Beach, FL), with Sequest search software used for peptide identification using the NCBI protein database.

### Immunocytochemistry and Confocal Microscopy

Cells were fixed in 4% paraformaldehyde, mounted in Vectashield medium containing DAPI for nuclear staining (Vector Laboratories, Burlingame, CA), and stained with β-catenin-AlexaFluor 488 (Cell Signaling) antibody, according to manufacturer’s protocol. Confocal fluorescence and two photon laser scanned images were taken on a Leica TCS-SP MP confocal microscope (Heidelberg, Germany).

### Cell Transfection and Topflash Luciferase Reporter Assay

Cells were transfected using the FuGENE6, according to manufacturer’s protocol (Roche). For the luciferase reporter assays, the µg ratio between the Topflash luciferase vector (obtained from R. Moon) and pRL-TK (Promega, Madison, WI) control *Renilla* vector was 3∶1. The luciferase activity was determined using a Dual-Luciferase Reporter Assay (Promega). The FGFR3 vectors and vectors carrying different LRP6 variants were described previously [14], [24], vector carrying HA-myr-AKT-GFP was obtained from J. Chung, vector carrying SRC-Y529F was obtained from Millipore, RasV12 and RafCAAX vectors were obtained from Clontech (Mountain View, CA). Plasmids expressing V5-tagged FGFR2, EGFR and TRKA were created by cloning full-length human FGFR2, EGFR and TRKA cDNA into pcDNA3.1. vector (Invitrogene). FGFR2 mutants were created by site-directed mutagenesis according to the manufacturer’s protocol (Stratagene, La Jolla, CA).

## Supporting Information

Figure S1 **FGF2 upregulates WNT/β-catenin signaling independent of the PI3K/AKT pathway.** (A) RCS cells were treated with FGF2 (10 ng/ml) for the indicated times and analyzed for activatory Ser473 phosphorylation of AKT by WB. (B) AKT was immunoprecipitated from FGF2-treated cells and used in a kinase assay with recombinant GSK3 as a substrate. C1, C2 - untreated controls, samples with omitted ATP serve as a negative control for the kinase assay. Note the slight FGF2-mediated activation of AKT determined by both Ser473 WB and a kinase assay (quantified by densitometry), with corresponding increase in AKT-mediated inhibitory phosphorylation of GSK3α/β at Ser21/9. (C) Cells were transfected with Topflash firefly luciferase (F-Luc) and control *Renilla* luciferase (*R*-Luc) vectors, pretreated with PI3K/AKT inhibitors (Wortmannin and LY294002) for 1 hour prior to FGF2 (10 ng/ml) and WNT3a (40 ng/ml), and analyzed for luciferase activity 20 hours later. Both inhibitors showed little effect on FGF2 and/or WNT3a-mediated Topflash activation, when compared with ERK pathway inhibition (Fig. 3C). (D) Cells were transfected with a constitutively-active (ca) AKT mutant fused with green fluorescent protein (GFP) and analyzed for both transgene expression and phosphorylated GSK3 48 hours later. (E) Cells were transfected with ca-AKT-GFP, GFP or an empty vector together with Topflash firefly luciferase (F-Luc) and control *Renilla* luciferase (*R*-Luc) vectors, treated with FGF2 and/or WNT3a, and analyzed for luciferase activity 20 hours later. Note the lack of ca-AKT-GFP effect on both FGF2 and WNT3a-mediated Topflash activation.
(PDF)

Figure S2 **Analysis FGFR3 and LRP6 transgene expression in RCS cells.** (A) Cells were transfected with high amounts of either V5-tagged LRP6 (upper panel) or FGFR3 (lower panel) vectors (6 µg of plasmid per 1×10^5^ cells), and analyzed for the indicated molecules 48 hours later. K508M - kinase inactive FGFR3 mutant. Note the high amounts of Ser1490 phosphorylation of wild-type LRP6, indicating its constitutive activation in an overexpressed state. Also note the ERK activation in cells overexpressing highly active FGFR3 mutant K650E (lower blot). (B) Cells were transfected with the indicated FGFR3 variant together with LRP6 (0.4 µg of FGFR3 vector + 2.4 µg of LRP6 vector per 1×10^5^ cells), Topflash firefly luciferase (F-Luc) and control *Renilla* luciferase (*R*-Luc) vectors, grown for 48 hours, analyzed for indicated molecules by WB, and used for one of the three Topflash experiments presented in (C). Spontaneous wt LRP6 phosphorylation, as well as ERK activation by FGFR3-K650E are detectable even at low amount of the transfected vectors (quantified by densitometry). (C) Cells were transfected with the indicated FGFR3 variant together with LRP6, Topflash luciferase (F-Luc) and control *Renilla* luciferase (*R*-Luc) vectors, grown for 48 hours, and analyzed for luciferase activity. Data represent an average from three independent experiments, with the indicated standard deviations (* *p*<0.001; Student’s *t*-test). K508M - kinase inactive FGFR3 mutant.
(PDF)

Figure S3 **Constitutively active forms of RAF, RAS and SRC signal via ERK/LRP6 pathway.** (A, D) RCS cells were transfected with empty plasmid or plasmid encoding RafCAAX, RAS-V12 or SRC-Y529F, grown for 48 hours, and analyzed for indicated molecules by WB. (B, E) Cells were transfected as indicated together with Topflash firefly luciferase (F-Luc) and control *Renilla* luciferase (*R*-Luc) vectors, grown for 24 hours, treated with U0126 (20 µM) one hour before WNT3a (40 ng/ml), and analyzed for luciferase activity 20 hours later. Data represent an average from three or four transfections (each measured twice). Potent Topflash activation mediated by RafCAAX, RAS-V12 or SRC-Y529F was significantly rescued by U0126 (* *p*<0.0001, # *p*<0.001; Student’s *t*-test). (C, F) Cells were transfected with RafCAAX, RAS-V12 or SRC-Y529F together with wt LRP6 or LRP6-5A mutant, Topflash luciferase (F-Luc) and control *Renilla* luciferase (*R*-Luc) vectors, grown for 48 hours, and analyzed for luciferase activity. Data represent an average from three or four transfections (each measured twice), with the indicated standard deviations. Statistically significant differences are indicated (* *p*<0.0001; Student’s *t*-test).
(PDF)

Figure S4 **Effect of activating FGFR3 mutants on basal levels of Topflash activity.** Cells were transfected with the indicated FGFR3 vectors together with Topflash firefly luciferase (F-Luc) and control *Renilla* luciferase (*R*-Luc) vectors, and analyzed for luciferase activity 48 hours later. Data represent an average from four transfections (each measured twice), with the indicated standard deviations (* *p*<0.001; Student’s *t*-test; compared to wt FGFR3). Results are representative of four experiments. Note the differences in the basal Topflash transactivation mediated by FGFR3 mutants, which correspond to relative levels of FGFR3 activation by each particular mutation [24].
(PDF)

Figure S5 **Effect of FGF signaling on WNT/β-catenin signaling in PC12 cells.** Cells were transfected with Topflash vector, the indicated FGFR3 expression plasmid, and *Renilla* luciferase vector at a ratio of 1∶1∶0.1. Cells were transfected with a total of 10.5 µg DNA using Lipofectamine 2000 (Invitrogen). The following day, cells were serum-starved 3 hours, treated with 40 ng/ml FGF1 and/or 20 ng/ml WNT3a, and analyzed for luciferase activity 24 hours later. F- FGF1, W - WNT3a, wild-type - wt FGFR3, kinase dead - K508M-FGFR3, K650E - K650E-FGFR3 mutant. Data represent three independent experiments. Statistically significant differences are indicated (Two-tailed T-test; * *p*<0.01, ** *p*<0.001).
(PDF)
