# Supplementary figures and images for: Receptor Tyrosine Kinases Activate Canonical WNT/β-Catenin Signaling via MAP Kinase/LRP6 Pathway and Direct β-Catenin Phosphorylation
Source: PLoS One. 2012 Apr 27;7(4):e35826. doi: 10.1371/journal.pone.0035826 (PMC3338780; doi:10.1371/journal.pone.0035826)

**FIGURE S1**

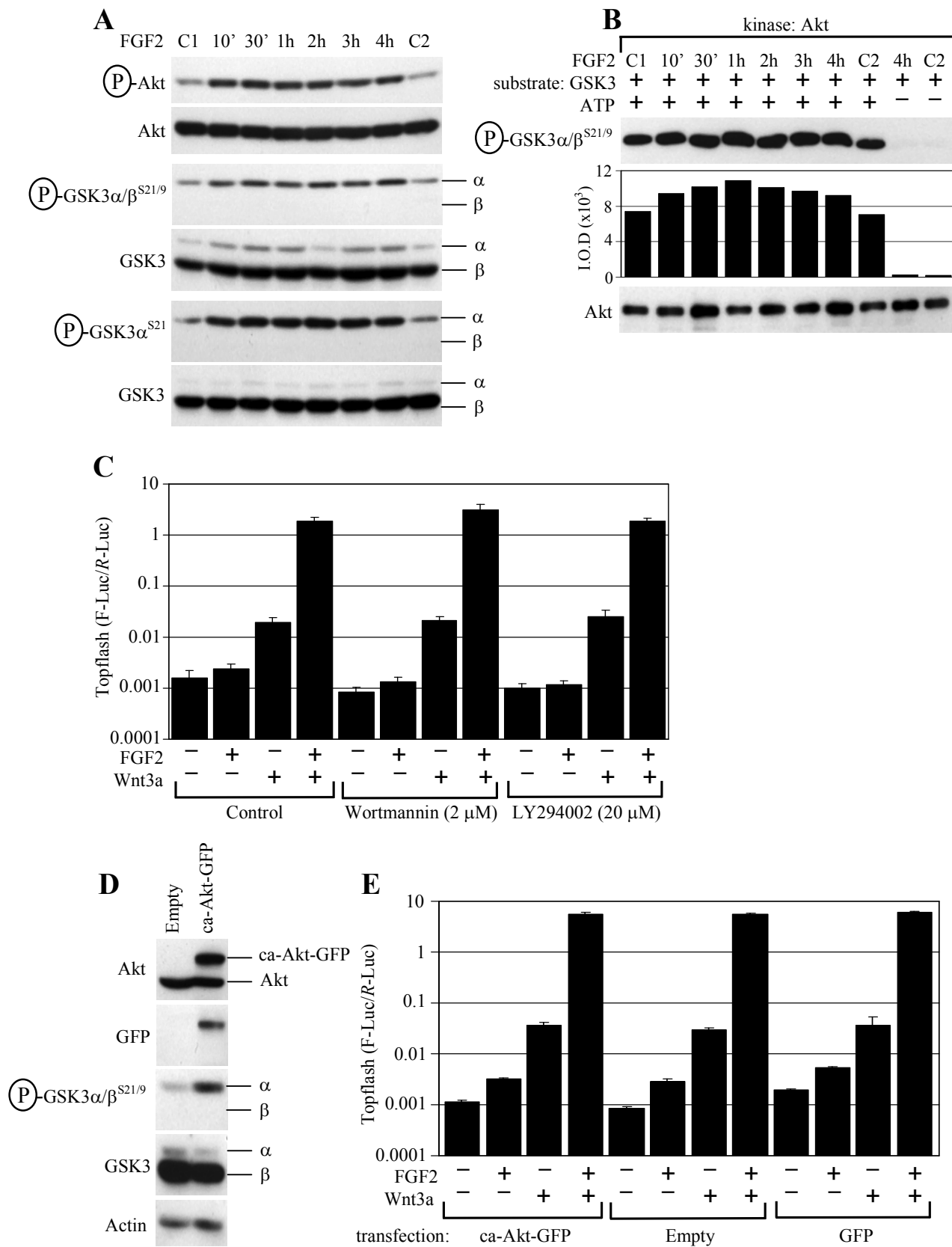

Supplement: Figure S1 — FGF2 upregulates WNT/β-catenin signaling independent of the PI3K/AKT pathway. (A) RCS cells were treated with FGF2 (10 ng/ml) for the indicated times and analyzed for activatory Ser473 phosphorylation of AKT by WB. (B) AKT was immunoprecipitated from FGF2-treated cells and used in a kinase assay with recombinant GSK3 as a substrate. C1, C2 - untreated controls, samples with omitted ATP serve as a negative control for the kinase assay. Note the slight FGF2-mediated activation of AKT determined by both Ser473 WB and a kinase assay (quantified by densitometry), with corresponding increase in AKT-mediated inhibitory phosphorylation of GSK3α/β at Ser21/9. (C) Cells were transfected with Topflash firefly luciferase (F-Luc) and control Renilla luciferase (R-Luc) vectors, pretreated with PI3K/AKT inhibitors (Wortmannin and LY294002) for 1 hour prior to FGF2 (10 ng/ml) and WNT3a (40 ng/ml), and analyzed for luciferase activity 20 hours later. Both inhibitors showed little effect on FGF2 and/or WNT3a-mediated Topflash activation, when compared with ERK pathway inhibition (Fig. 3C). (D) Cells were transfected with a constitutively-active (ca) AKT mutant fused with green fluorescent protein (GFP) and analyzed for both transgene expression and phosphorylated GSK3 48 hours later. (E) Cells were transfected with ca-AKT-GFP, GFP or an empty vector together with Topflash firefly luciferase (F-Luc) and control Renilla luciferase (R-Luc) vectors, treated with FGF2 and/or WNT3a, and analyzed for luciferase activity 20 hours later. Note the lack of ca-AKT-GFP effect on both FGF2 and WNT3a-mediated Topflash activation. (PDF) [file pone.0035826.s001.pdf]

**FIGURE S2**

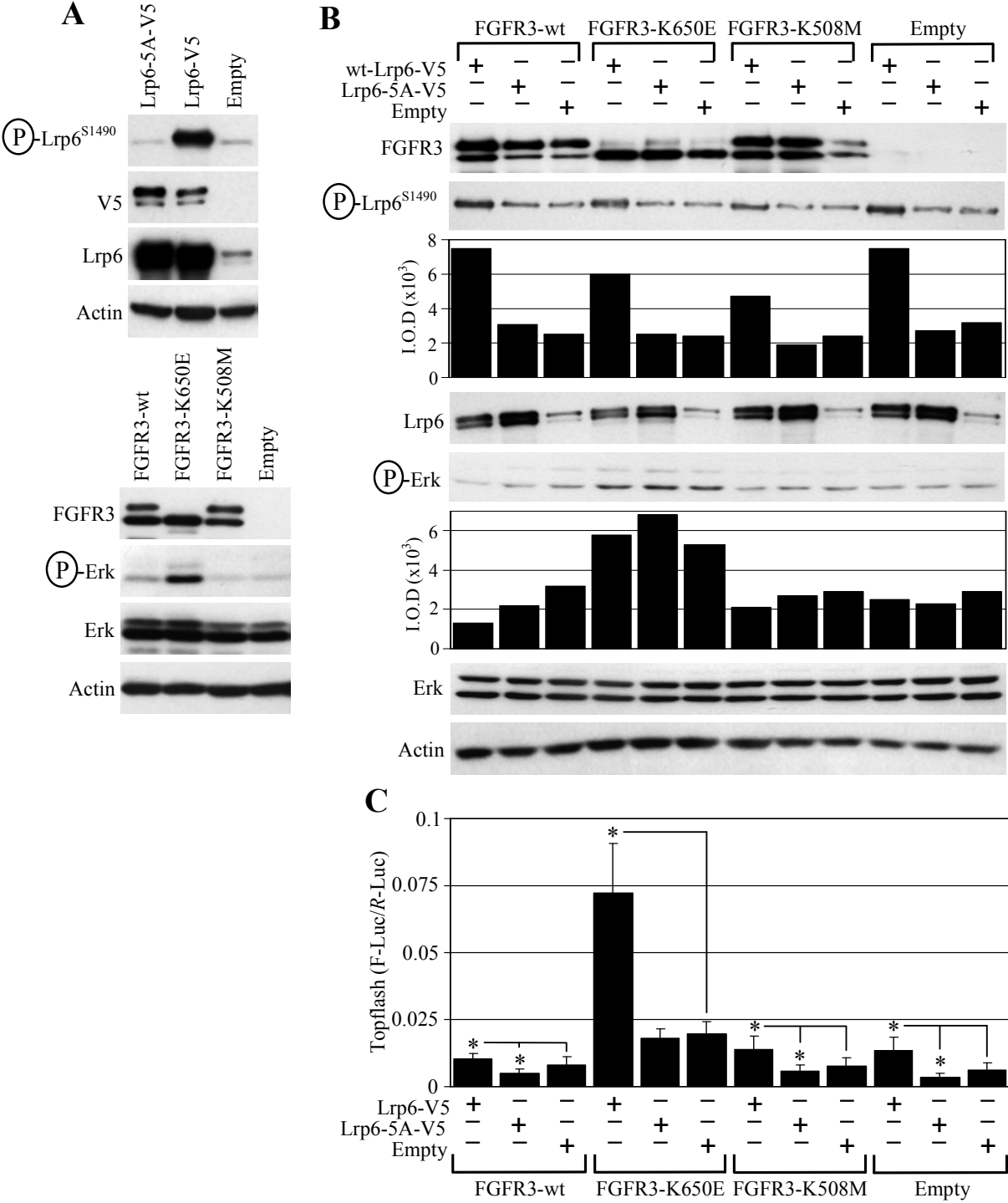

Supplement: Figure S2 — Analysis FGFR3 and LRP6 transgene expression in RCS cells. (A) Cells were transfected with high amounts of either V5-tagged LRP6 (upper panel) or FGFR3 (lower panel) vectors (6 µg of plasmid per 1×10^5^ cells), and analyzed for the indicated molecules 48 hours later. K508M - kinase inactive FGFR3 mutant. Note the high amounts of Ser1490 phosphorylation of wild-type LRP6, indicating its constitutive activation in an overexpressed state. Also note the ERK activation in cells overexpressing highly active FGFR3 mutant K650E (lower blot). (B) Cells were transfected with the indicated FGFR3 variant together with LRP6 (0.4 µg of FGFR3 vector + 2.4 µg of LRP6 vector per 1×10^5^ cells), Topflash firefly luciferase (F-Luc) and control Renilla luciferase (R-Luc) vectors, grown for 48 hours, analyzed for indicated molecules by WB, and used for one of the three Topflash experiments presented in (C). Spontaneous wt LRP6 phosphorylation, as well as ERK activation by FGFR3-K650E are detectable even at low amount of the transfected vectors (quantified by densitometry). (C) Cells were transfected with the indicated FGFR3 variant together with LRP6, Topflash luciferase (F-Luc) and control Renilla luciferase (R-Luc) vectors, grown for 48 hours, and analyzed for luciferase activity. Data represent an average from three independent experiments, with the indicated standard deviations (* p<0.001; Student’s t-test). K508M - kinase inactive FGFR3 mutant. (PDF) [file pone.0035826.s002.pdf]

**FIGURE S3**

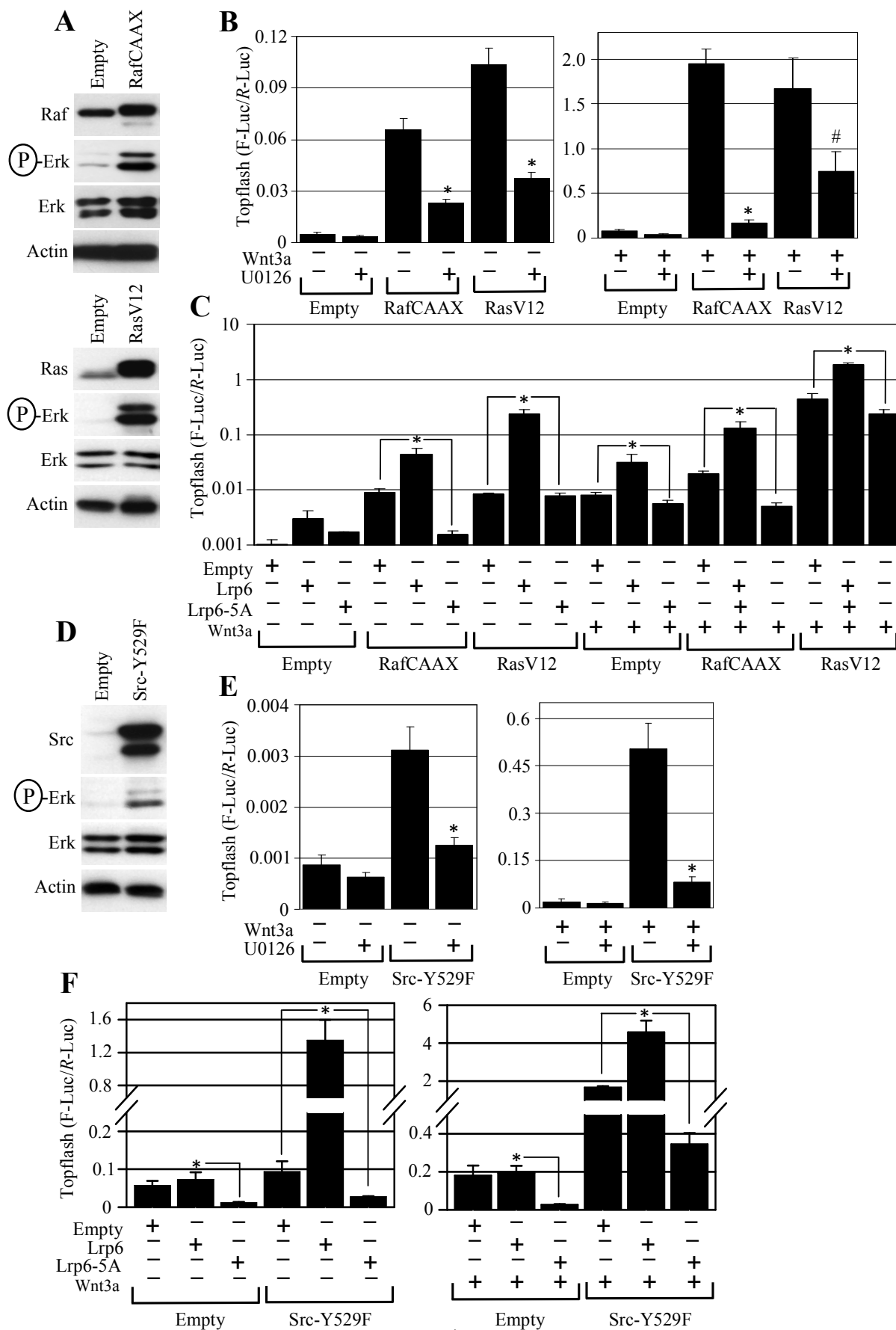

Supplement: Figure S3 — Constitutively active forms of RAF, RAS and SRC signal via ERK/LRP6 pathway. (A, D) RCS cells were transfected with empty plasmid or plasmid encoding RafCAAX, RAS-V12 or SRC-Y529F, grown for 48 hours, and analyzed for indicated molecules by WB. (B, E) Cells were transfected as indicated together with Topflash firefly luciferase (F-Luc) and control Renilla luciferase (R-Luc) vectors, grown for 24 hours, treated with U0126 (20 µM) one hour before WNT3a (40 ng/ml), and analyzed for luciferase activity 20 hours later. Data represent an average from three or four transfections (each measured twice). Potent Topflash activation mediated by RafCAAX, RAS-V12 or SRC-Y529F was significantly rescued by U0126 (* p<0.0001, # p<0.001; Student’s t-test). (C, F) Cells were transfected with RafCAAX, RAS-V12 or SRC-Y529F together with wt LRP6 or LRP6-5A mutant, Topflash luciferase (F-Luc) and control Renilla luciferase (R-Luc) vectors, grown for 48 hours, and analyzed for luciferase activity. Data represent an average from three or four transfections (each measured twice), with the indicated standard deviations. Statistically significant differences are indicated (* p<0.0001; Student’s t-test). (PDF) [file pone.0035826.s003.pdf]

**FIGURE S4**

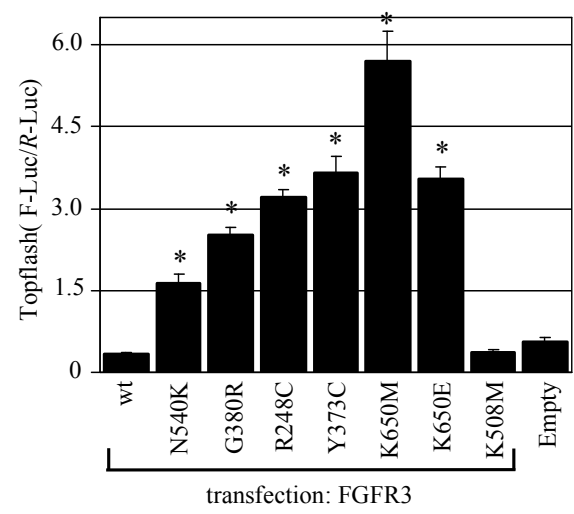

Supplement: Figure S4 — Effect of activating FGFR3 mutants on basal levels of Topflash activity. Cells were transfected with the indicated FGFR3 vectors together with Topflash firefly luciferase (F-Luc) and control Renilla luciferase (R-Luc) vectors, and analyzed for luciferase activity 48 hours later. Data represent an average from four transfections (each measured twice), with the indicated standard deviations (* p<0.001; Student’s t-test; compared to wt FGFR3). Results are representative of four experiments. Note the differences in the basal Topflash transactivation mediated by FGFR3 mutants, which correspond to relative levels of FGFR3 activation by each particular mutation [24]. (PDF) [file pone.0035826.s004.pdf]

FIGURE S5

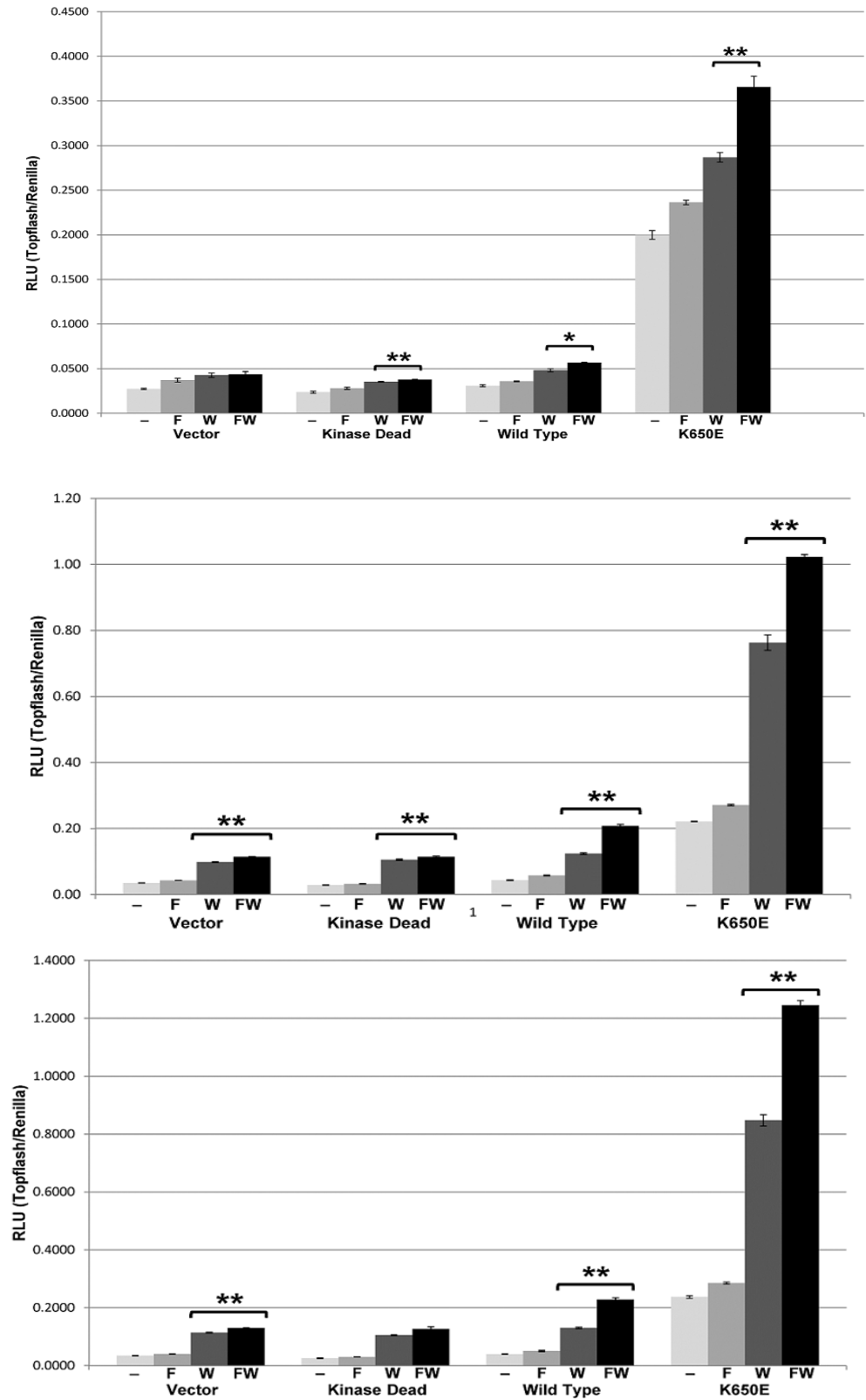

Supplement: Figure S5 — Effect of FGF signaling on WNT/β-catenin signaling in PC12 cells. Cells were transfected with Topflash vector, the indicated FGFR3 expression plasmid, and Renilla luciferase vector at a ratio of 1∶1∶0.1. Cells were transfected with a total of 10.5 µg DNA using Lipofectamine 2000 (Invitrogen). The following day, cells were serum-starved 3 hours, treated with 40 ng/ml FGF1 and/or 20 ng/ml WNT3a, and analyzed for luciferase activity 24 hours later. F- FGF1, W - WNT3a, wild-type - wt FGFR3, kinase dead - K508M-FGFR3, K650E - K650E-FGFR3 mutant. Data represent three independent experiments. Statistically significant differences are indicated (Two-tailed T-test; * p<0.01, ** p<0.001). (PDF) [file pone.0035826.s005.pdf]
